# Classification of Plant Leaves Using New Compact Convolutional Neural Network Models

**DOI:** 10.3390/plants11010024

**Published:** 2021-12-22

**Authors:** Shivali Amit Wagle, R. Harikrishnan, Sawal Hamid Md Ali, Mohammad Faseehuddin

**Affiliations:** 1 E&TC Department, Symbiosis Institute of Technology, Symbiosis International Deemed University, Pune 412115, India; kulkarni_shivali@yahoo.co.in (S.A.W.); faseehuddin03@gmail.com (M.F.); 2 Department of Electrical, Electronic and Systems Engineering, Universiti Kebangsaan Malaysia, Bangi 43600, Malaysia

**Keywords:** classification, compact model, convolutional neural network, plant leaf

## Abstract

Precision crop safety relies on automated systems for detecting and classifying plants. This work proposes the detection and classification of nine species of plants of the PlantVillage dataset using the proposed developed compact convolutional neural networks and AlexNet with transfer learning. The models are trained using plant leaf data with different data augmentations. The data augmentation shows a significant improvement in classification accuracy. The proposed models are also used for the classification of 32 classes of the Flavia dataset. The proposed developed N1 model has a classification accuracy of 99.45%, N2 model has a classification accuracy of 99.65%, N3 model has a classification accuracy of 99.55%, and AlexNet has a classification accuracy of 99.73% for the PlantVillage dataset. In comparison to AlexNet, the proposed models are compact and need less training time. The proposed N1 model takes 34.58%, the proposed N2 model takes 18.25%, and the N3 model takes 20.23% less training time than AlexNet. The N1 model and N3 models are size 14.8 MB making it 92.67% compact, and the N2 model is 29.7 MB which makes it 85.29% compact as compared to AlexNet. The proposed models are giving good accuracy in classifying plant leaf, as well as diseases in tomato plant leaves.

## 1. Introduction

Plants provide food for all living things, making them the backbone of the ecosystem [1]. Plant species are valuable as medicine, foodstuff, and also for industrial applications. Some plants are at risk of extinction. So, it is imperative to set up a database for plant protection. Manual examination of the plant with the naked eye is the most basic or conventional technique. This procedure, involves constant supervision of a wide range of farm areas by experts [2,3]. This is a time-consuming and costly procedure. To achieve plant protection, the classification of the plant plays an essential role [4]. Plants leaves are easily accessible and prominent parts, unlike flowers which are available for a shorter period. The leaves are, therefore, a good choice for automatic plant classification. The leaves are important in exploring the genetic relationship of plants and the explanation of plant development. However, given a large number of species, plant identification, even for botanists, is a relatively difficult task [5,6]. The leaf recognition technology was followed by botanists for classifying specific plant species. Plants generally have distinctive features that differ in many aspects, such as texture, shape, color, and size; they are different [7]. In the last couple of years, different “Computer-Aided Detection” (CAD) methods are deployed for leaf based plant recognition owing to their high classification accuracy [8,9].

The interdisciplinary approach of plant classification combines botanical data, and the concept of species with computer solutions [10]. Recent advances in science and technology allow computer vision approach to help botanists to identify plants. Computer vision researchers have used leaves to classify plants as a comparative tool [11]. From the machine learning point of view, the classification problem can be addressed by adopting a new quick solution, which will bring experts, farmers, decision-makers, and strategists into a single chorus [12].

In recent years, evolutionary neural networks have attracted much attention of the researchers because of their ability to give superior image classification accuracy. They combine the neural network and computation to solve any problem. Krizhevsky et al. [13] set a record of 10.9% more classification accuracy compared to the second-best entry in ImageNet in the 2012 Large Scaled Challenge for Visual Recognition. Advances in the processing of images provided various preprocessing techniques for the extraction of images. Feature extraction is the step taken to identify discriminatory characteristics that form the basis for classification. The classification task can be performed with multiple learning technologies such as “Support Vector Machines” (SVM), Naïve Baye, “K-Nearest Neighbor” (KNN), and “Convolutional Neural Network” (CNN) [14].

Deep learning is a subset of machine learning that consists of a set of algorithms for modeling high-level data abstractions using a deep graph with multiple processing layers that include linear and non-linear transformations [15]. CNNs are well-suited for image classification tasks due to their close relationship between layers and spatial information, which explains their popularity in recent plant classifiers.

Several studies have found that image-based assessment methods produce more accurate and consistent results than human visual assessments [16]. A lot of work has been completed to classify things using various techniques. Lecun et al. [17] introduced the basic deep learning tool of CNN as an introduction to deep learning model techniques in the field of classification and detection. In recent years, deep learning models have been used to a small extent in agriculture. CNNs are a form of a dynamic model that aids classification applications. For classification, there are many CNN models, such as AlexNet [13], GoogLeNet [18], ResNet50, ResNet18, ResNet101 [19], VGG16, VGG19 [20], DenseNet [21], SqueezeNet [22], and others.

Mohanty et al. [23] classified 14 different plant leaves using AlexNet and GoogLeNet, with an accuracy of 99.27% and 99.34%, respectively. The authors used different input data, such as color images, segmented images, and grayscale images separately. Dyrmann et al. [24] classified the plant leaf data with a CNN model and achieved an accuracy of 86.2%. Barré et al. [25] in their work for plant leaf classification, used LeafSnap, Foliage, and Flavia dataset for classification of different classes with their proposed model LeafNet. A total of 184 classes of LeafSnap were classified with an accuracy of 86.3%, and 60 classes of the Foliage dataset were classified with an accuracy of 95.8%. They achieved the performance accuracy of 97.9% for the Flavia dataset with 32 classes. A deep CNN model [10] with “Multilayer Perceptron” (MLP) classifier achieved 97.7% accuracy and improved to 98.1% accuracy with SVM classifier for the MalayaKew dataset with 44 classes. Haque et al. [26] have presented work for plant classification that uses geometric features in preprocessing and achieved an accuracy of 90% for the classification of 10 plant species of Flavia dataset. Gao et al. [1] achieved an accuracy of 84.2% in a LifeCLEF Plant Identification Task with their proposed 3SN Siamese network that learns from spatial and structural features for the leaf classification task. The recognition of plant family and further identifying the plant class for the four datasets was performed with two ways attention CNN model by [27].

In the preparation of Ayurvedic medicines, the identification and classification of medicinal plants play an essential role. In addition, it is important for farmers, botanists, practitioners, the forest department’s offices, and those involved in the preparation of Ayurvedic medicines for a correct classification of medicinal plants. Medicinal plant classification by [28] with AlexNet model achieved an accuracy of 94.87%, and for the Ayurleaf CNN model, the accuracy is 95.06%. Duong-Trung et al. [12] achieved 98.5% classification accuracy with the MobileNet model for 20 species of self-collected medicinal plant data.

Liu et al. [29] proposed a ten-layer CNN model for the classification of plant leaf and achieved an accuracy of 87.92% for the 32 classes. ResNet model gave the classification accuracy of 93.09% for plant identification with LeafSnap dataset [9]. Plant leaf classification was completed by [5] on the images captured by Silva et al. [30] using an Apple iPad device. The Deep Neural network (DNN) model shows 91.17% accuracy, and with the CNN model, the accuracy is improved to 95.58%. The classification of plant leaf with the complex background was completed by [6] on the images captured through mobile phones. The classification accuracy is 91.5%, 92.4%, and 89.6% for VGG16, VGG 19, and the Inception ResNetV2 model, respectively. For the identification of the berry plants, Ref. [14] used the AlexNet model and achieved an accuracy of 97.80% for the three classes of self-collected data of berry plants. A comparative analysis of the work related to the classification of plants is shown in Table 1.

Once the classification of the plant is completed, further work can be extended to the classification of disease. The VGG16 model was trained with transfer learning for the apple leaf disease and yielded an overall accuracy of 90.4% [16]. With the augmented dataset of 14828 images of tomato leaves, Ref. [31] achieved an accuracy of 98.66% for AlexNet and 98.18% for the VGG16 model. A small CNN was proposed by [32] for the classification of the plant into a healthy or diseased category and achieved an accuracy of 96.6%. The classification accuracy achieved by [33] for tomato plant disease with the laboratory data is 98.50% for the VGG16 model, 98.30% for the VGG19 model, 99.40%for ResNet model, and 99.60% for the Inception V3 model. The proposed model of [34] outperformed AlexNet and VGG16 with an accuracy of 99.45% in the classification of tomato plant leaf, with an accuracy of 90.1%.

Guava fruit diseases classification by [35] achieved an accuracy of 99% for the Bagged Tree classifier on a set of RGB, HSV, and LBP features. Detection of Cassava plant disease by [36] achieved an accuracy of 96.75% with the deep residual neural network model. Alli et al. [37] used a data augmentation method to achieve an accuracy of 99.7% for cassava plant disease classification using MobileNetV2. Pearl millet disease classification with an automated method of collecting the pearl millet data from the farm and classifying the disease with a Custom-Net model with an accuracy of 98.78% [38]. A comparative analysis of the work related to plant disease classification is shown in Table 2.

In this work, the proposed CNN models are used for the classification of plant species for the PlantVillage (PV) and Flavia datasets. The performance of the developed models are compared with the AlexNet model with transfer learning. The proposed models have less depth as compared to AlexNet. The proposed models are compact in size and require less training time, maintaining good accuracy. The main contributions of this work are as follows:

1. Three highly accurate and compact models namely, N1, N2, and N3 are proposed for plant leaves classification. The proposed models show high classification accuracy, and they require less training time;
2. The performance of the models is validated by employing them to classify leaves from challenging PV and Flavia datasets. The models exhibit high classification accuracy;
3. To validate the versatility of the proposed models, they are also employed in tomato leaves disease classification using images captured from mobile phone. The disease classification accuracy shows that the proposed models are well suited for both plant leaves classification and disease classification.

## 2. Material and Methods

**Figure 1:**
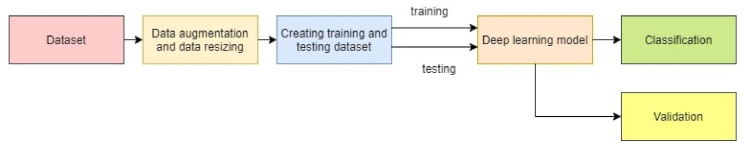
Proposed workflow for classification and validation of plant.

The work discusses the classification of a plant with a newly developed compact CNN model and AlexNet with transfer learning. The nine classes belonging to nine different species of plant images of the PlantVillage database are used for the classification. Additionally, the 32 classes of the Flavia dataset are classified. Figure 1 shows the workflow for plant classification and validation. The PV and Flavia datasets are augmented separately, and images are resized to a required size. The input image size for the proposed models is 256 × 256 × 3, and the input image size for AlexNet is 227 × 227 × 3. The dataset is further split into 80–20% of training dataset and testing dataset. The proposed models and AlexNet are trained with the training dataset for the classification of the plant species. The trained model is used for validation with the testing data for the prediction of the new data class.

### 2.1. Dataset of Plant Leaves

A PV dataset includes images of healthy and diseased leaves of 38 different classes [23]. The dataset includes both healthy and diseased leaf categories of nine plant species, which include “apple, cherry, corn, grapes, peach, pepper, potato, strawberry, and a tomato” plant. We are considering the plant species here for classification purposes. The Flavia dataset [4] with 32 classes was used for classification with the proposed models. The Flavia dataset consists of a variant class of the plant leaf belonging to different crops like “Anhui Barberry”, “Beale’s barberry”, “Big-fruited Holly”, “Camphortree”, “Canadian Poplar”, “Castor Aralia”, “Chinese Cinnamon”, “Chinese Horse Chestnut”, “Chinese Redbud”, “Chinese Toon”, “Chinese Tulip Tree”, “Crape Myrtle” or “Crepe Myrtle”, “Deodar”, “Ford Woodlotus”, “Ginkgo Maidenhair Tree”, “Glossy Privet”, “Goldenrain Tree”, “Japan Arrowwood”, “Japanese Cheesewood”, “Japanese Flowering Cherry”, “Japanese Maple”, “Nanmu”, “Oleander”, “Peach”, “Pubescent Bamboo”, “Southern Magnolia”, “Sweet Osmanthus”, “Tangerine”, “Trident Maple”, “True Indigo”, “Wintersweet”, “Yew Plum Pine”.

### 2.2. Dataset Pre-Processing

Pre-processing the data is essential to maintain the uniformity and smooth functioning of the algorithm [24]. Deep learning behaves well when the input dataset is as large as possible and avoids overfitting. The very minute, invisible to human eye changes, such as adding noise and blur to the input images, can help CNNs learn more robust features [39,40]. In this work, the dataset is augmented with the Gaussian blur, salt and pepper noise with randomized scaling of 0.95 to 1.05 in a horizontal and vertical direction, and random rotation in the range −30° to 30° of the images. The combination of augmentation used is shown in Table 3. The other augmentation performed here is with the rotation and flipping of the dataset. In position augmentation, images are rotated by 45°, 135°, 225°, and 315° and flipped horizontally and vertically. The color augmentation of saturation, hue, and contrast is added. Saturation represents the amount of purity or colorfulness of a color. Hue represents the color (blue, red, green, etc.); its value ranges from 0 to 360. Histogram equalization is performed in evaluating the contrast value in color augmentation. Histogram equalization is known to improve accuracy.

The classification of plant leaves data is completed on a dataset with 38,400 images and an augmented dataset of 336,000 images. The deep learning network used in this work is proposed CNN model 1 (N1 model), CNN model 2 (N2 model), CNN model 3 (N3 model), and AlexNet model with transfer learning. The input images are resized to the size 256 × 256 × 3 for the proposed developed models, and the images are resized to 227 × 227 × 3 for the AlexNet model.

### 2.3. Deep Learning Model

**Figure 2:**
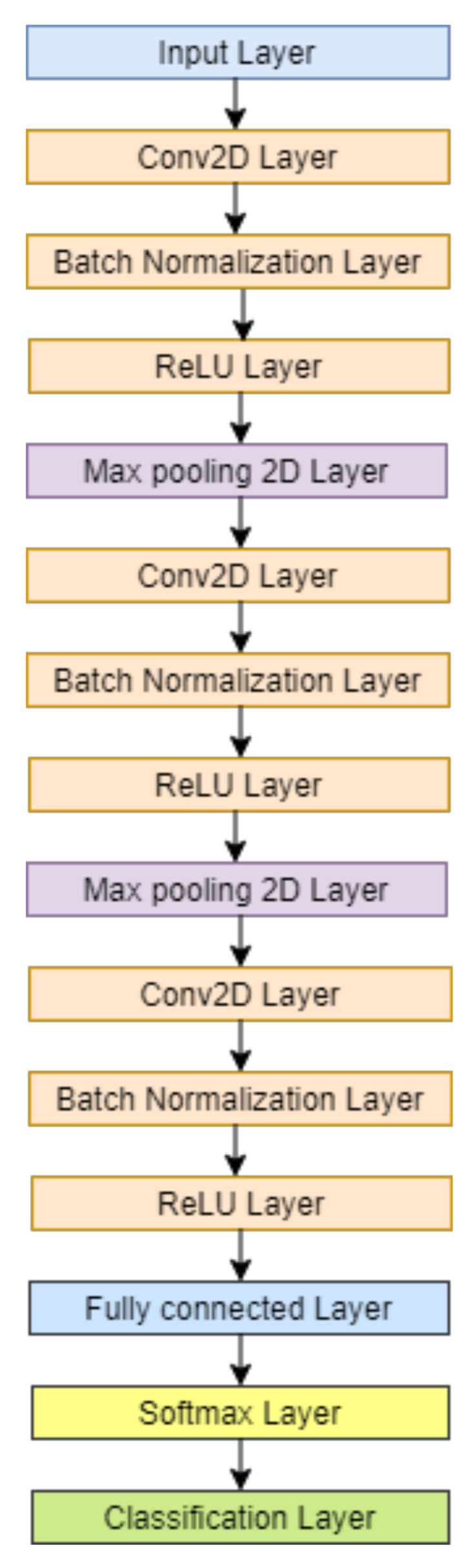
Proposed compact CNN model for classification and validation.

The aim of this analysis is to create a computationally compact and accurate learning model for plant leaf classification. The proposed CNN model used in this work is developed with three convolutional layers as shown in Figure 2. The model consists of three sets of convolution 2D layer (Conv2D) followed by batch normalization layer and ReLU layer. There are three sets of Conv2D layer, batch normalization layer, and ReLU layer. The first two sets are followed by the max-pooling layer, and the third set is followed by the fully connected layer, softmax classifier, and classification layer. The convolution layer is modified with the size of the filter and the number of filters in the three CNN models of N1, N2, and N3, as shown in Table 4.

The convolutional layer specifies a set of filters that perform convolution across the entire image. Each convolutional layer in this architecture learns the various attributes that capture discriminatory patterns to differentiate the type of plant leaf. After each gradient update on a batch of data, Deep Neural Networks see different feature information from the previous layer. Furthermore, because the parameters of the previous layers are updated during the training phase, the data distribution of this input feature map varies greatly. This has a significant impact on training speed and necessitates the use of various heuristics to determine parameter initialization. The Rectified Linear Unit (ReLU) is an activation function commonly used in the design of neural networks, particularly CNNs. It is the identity function, f(x) = x, for all positive values of input ‘x’, and zeros out for negative values. ReLU is activated infrequently, mimicking the biological neuron’s inactivity in response to certain impulses. This max-pooling layer only activates a subset of the neurons in the feature map. It is used across all blocks on a ‘2-by-2’ window with a stride factor of ‘2’. The feature maps’ width and height are effectively reduced while the number of channels remains constant. In CNN models that predict a multinomial probability distribution, the softmax function is used as the activation function in the output layer. In other words, for multi-class classification problems, softmax is used as the classifier.

One of the benefits of small filter sizes over fully connected networks is that they minimize computing costs and weight sharing, resulting in lower back-propagation weights. Until now, the best choice for practitioners has been 3 × 3 [41,42]. The CNN model N1 has a fixed filter size of 3 × 3 in all three convolution layers. In the 1st Conv2D, there are eight filters and, in the 2nd, Conv2D and 3rd Conv2D, there are 16 and 32 filters, respectively. In the CNN model N2, the filter size is kept the same as N1, but the number of filters in them is doubled as compared to N1. In the CNN model of N3, the filter size for the 1st Conv2D layer is 7 × 7, with eight filters. The 2nd Conv2D layer is 5 × 5, with 16 filters, and the 3rd Conv2D layer is 3 × 3 with 32 filters. The 1st Conv2D layer and 2nd Conv2D layer is followed by a max-pooling layer with a stride of 2 on a 2-by-2 window. The dataset is divided into training and testing datasets with the combination of 80–20% of the total data of 38,400 and 336,000 images. The data are trained with this combination for all the CNN models for the classification of the plant leaves. AlexNet is a pre-trained model that has the ability to classify up to 1000 classes [13]. In this work, we are classifying plant leaves of PV and Flavia dataset with 9 and 32 classes, respectively. For this purpose, AlexNet with transfer learning is used for classification. The objective of transfer learning is to optimise learning by leveraging the transferability of knowledge from the source [12]. All the models are implemented using the deep learning toolbox of MATLAB2019b in this study.

### 2.4. Performance Parameters of the CNN Model

The classification of the deep learning models is based on the performance and accuracy of the model. The confusion matrix of the test dataset is used for evaluating the performance parameters. The correct classification is shown by the diagonal elements and misclassification by non-diagonal elements of the confusion matrix. The elements of the confusion matrix are as follows [43]:

- “True Positive (TP): is the correctly labeled positive samples by the classifier”;
- “True Negative (TN): is the correctly labeled negative samples by the classifier”;
- “False Positive (FP): is the negative samples incorrectly labeled as positive”; and
- “False Negative (FN): is the positive samples incorrectly labeled as negative”.

The performance parameters evaluated here are macro recall, macro precision, macro F1 score, and mean accuracy [31]. Sensitivity/recall is the measure of the model that appropriately detects the positive class and is also known as the true positive rate. The model assigning positive events to the positive class is measured by a positive predictive value, also known as precision. F1 score is the harmonic mean of recall and precision. “Macro recall is the average per class effectiveness of a classifier to identify class labels”. “Macro precision is an average per class agreement of the data class labels with those of the classifiers”. “Macro F1 score is the relation between data’s positive labels and those given by the classifier based on per class average”. “Accuracy is the ratio of correct prediction by all predictions”.

(1) $$Sensitivity/Recall=\frac{TP}{TP+FN}.$$

(2) $$MacroRecall=\frac{\sum_{n=1}^{C}Sensitivity}{C}$$

where *C* is the number of classes.

(3) $$Precision=\frac{TP}{TP+FP}$$

(4) $$MacroPrecision=\frac{\sum_{n=1}^{C}Precision}{C}$$

(5) $$F1score=\frac{2\times Precision\times Recall}{Precision+Recall}$$

(6) $$MacroF1score=\frac{\sum_{n=1}^{C}F1score}{C}$$

(7) $$Accuracy=\frac{TP+TN}{TP+TN+FP+FN}$$

There are nine classes in the PV dataset, so the confusion matrix metrics 9 × 9. In the case of the Flavia dataset with 32 classes, the confusion matrix metrics 32 × 32. For the N1 model, N2 model, N3 model, and AlexNet model, the accuracy of each class is evaluated. Each deep learning model’s simulation time is noted. The time is in seconds, measured.

### 2.5. Validation of the Trained CNN Model

Validation of models is completed for the trained CNN models with the images from the PV and Flavia datasets, respectively, that was not part of the training or testing set. The validation of models is performed with 33,600 images. The validation of the model classifies the unknown image leaf data with its class and accuracy.

## 3. Results and Discussion

**Figure 3:**
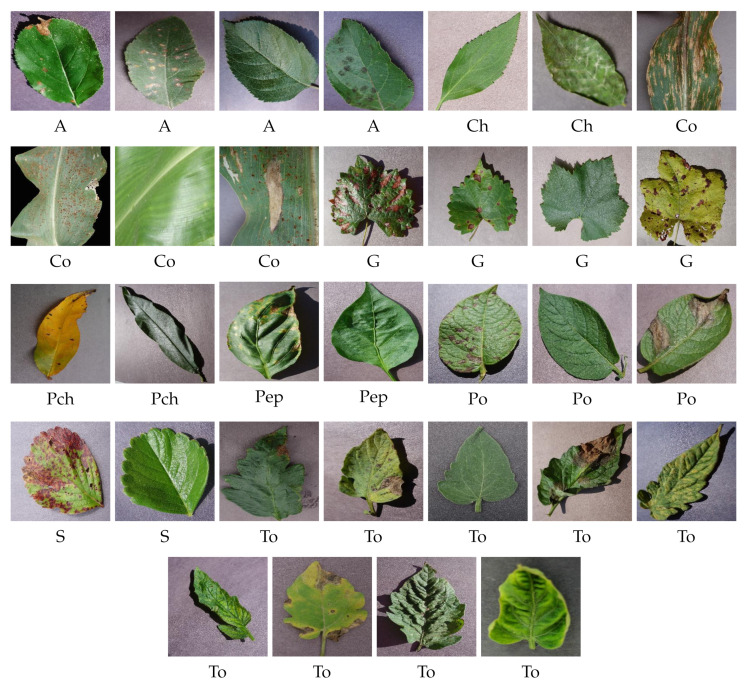
Plant leaf images from the training dataset of PV dataset.

The PV dataset with nine species of plants is shown in Figure 3. The classes are abbreviated as follows. Apple plant with four varieties are A; Cherry with two varieties are Ch. Corn with four varieties are Co; Grape with four varieties are G. Peach with two varieties are Pch; Pepper with two varieties are Pep; potato with three varieties is Po. Strawberry with two varieties are S; tomato with nine varieties are To.

**Figure 4:**
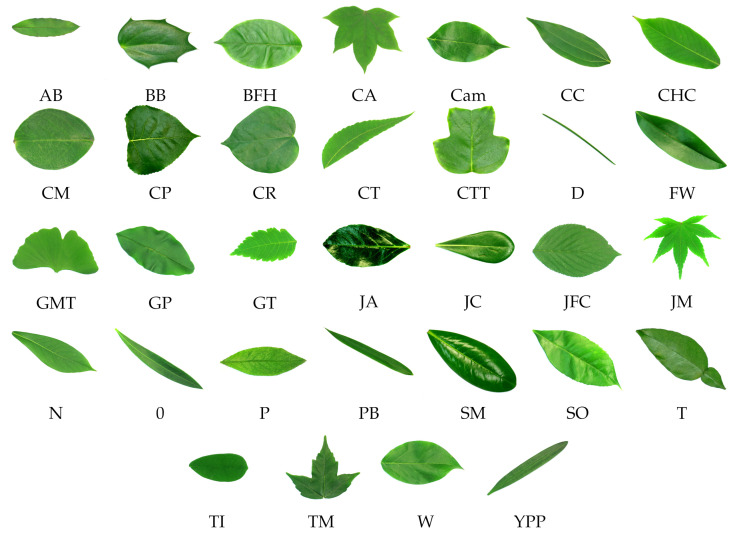
Plant leaf images from the training dataset of Flavia dataset.

The 32 classes of the Flavia dataset are shown in Figure 4. The classes are abbreviated as “Anhui Barberry” is AB, “Beale’s Barberry” is BB, “Big-Fruited Holly” is BFH, “Castor Aralia” is CA, “Camphortree” is Cam, “Chinese Cinnamon” is CC, “Chinese Horse Chestnut” is CHC, “Crape Myrtle” is CM, “Canadian Poplar” is CP, “Chinese Redbud’ is CR, “Chinese Toon” is CT, “Chinese Tulip Tree” is CTT, “Deodar” is D, “Ford Woodlotus” is FW, “Ginkgo Maidenhair Tree” is GMT, “Glossy Privet” is GP, “Goldenrain Tree” is GT, “Japan Arrowwood” is JA, “Japanese Cheesewood” is JC, “Japanese Flowering Cherry” is JFC, “Japanese Maple” is JM, “Nanmu” is N, “Oleander” is O, “Peach” is P, “Pubescent Bamboo” is PB, “Southern Magnolia” is SM, “Sweet Osmanthus” is SO, “Tangerine” is T, “Trident Maple” is TM, “True Indigo” is TI, “Wintersweet” is W, and “Yew Plum Pine” is YPP. All the 32 classes belong to different species here.

**Figure 5:**
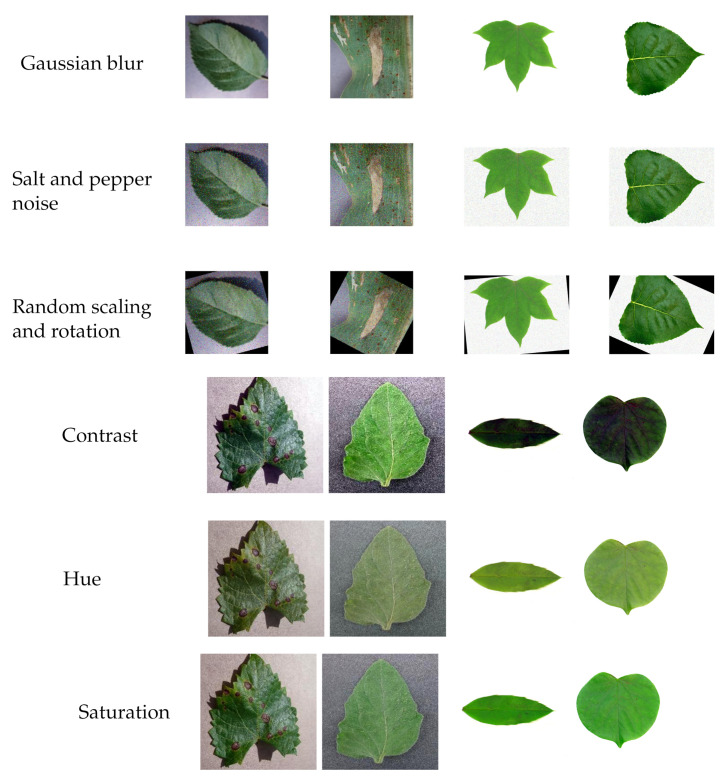
Some of the pre-processed images of PV and Flavia dataset.

The pre-processing of the dataset is discussed in Section 2.2. The dataset is augmented with augmented data 1 (ad1) and augmented data 2 (ad2) and further resized to 256 × 256 × 3 for the proposed models and 227 × 227 × 3 for the AlexNet. Some of the data augmented images are shown in Figure 5.

**Figure 6:**
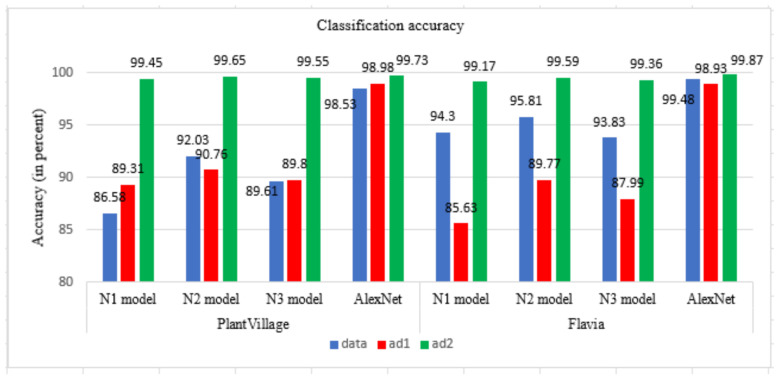
Classification accuracy of models for dataset, ad1 and ad2 for PV and Flavia dataset.

**Figure 7:**
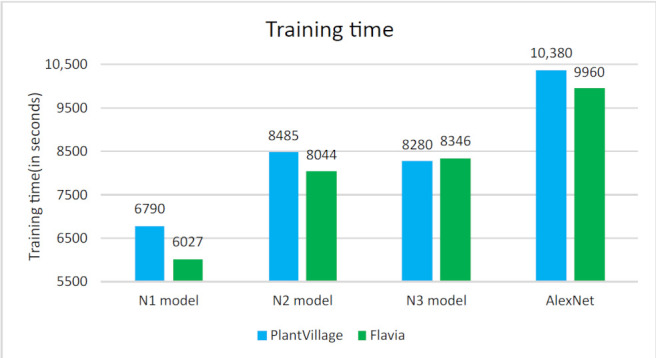
Training time of models for PV and Flavia dataset.

Classification of leaves of plants is performed using proposed compact models N1, N2, N3, and AlexNet with transfer learning. The classification accuracy of these models on a dataset, ad1, and ad2 is shown in Figure 6. The classification accuracy increases with the augmented dataset. More features are studied in the ad2, along with the increase in the number of images that are used for training the models. This helps in learning the model and achieving better prediction in terms of accuracy. The accuracy of the proposed N1 model is 86.58% with dataset and increased to 89.31% with ad1 and 99.45% with ad2. The accuracy of the proposed N2 model is 92.09% with the dataset and increased to 99.65% with ad2. The accuracy of the proposed N3 model is 89.61% and increases to 89.8% with ad1 and 99.55% with ad2. AlexNet shows an accuracy of 98.53% with a dataset and increases to 99.73% with ad2. The accuracy of the N1 model, N2 model, N3 model, and AlexNet is almost the same for ad2. The time for training the model increases as the number of images increase. The training time for the N1 model, N2 model, N3 model, and AlexNet model is shown in Figure 7. The proposed N1, N2, and N3 models take less training time as compared to the AlexNet model. The number of layers and the size of the filter used in the proposed CNN N1 model, N2 model, and N3 model is less than the traditional AlexNet model. There are three CNN layers in the proposed developed model, whereas there are five Convolutional layers in AlexNet. The filter size is also more in AlexNet as compared to proposed developed models. The number of CNN layers and the size of the filters used in our developed model is compact as compared to AlexNet. This reduces the complexity of the model and so the training time required for the model is less.

**Figure 8:**
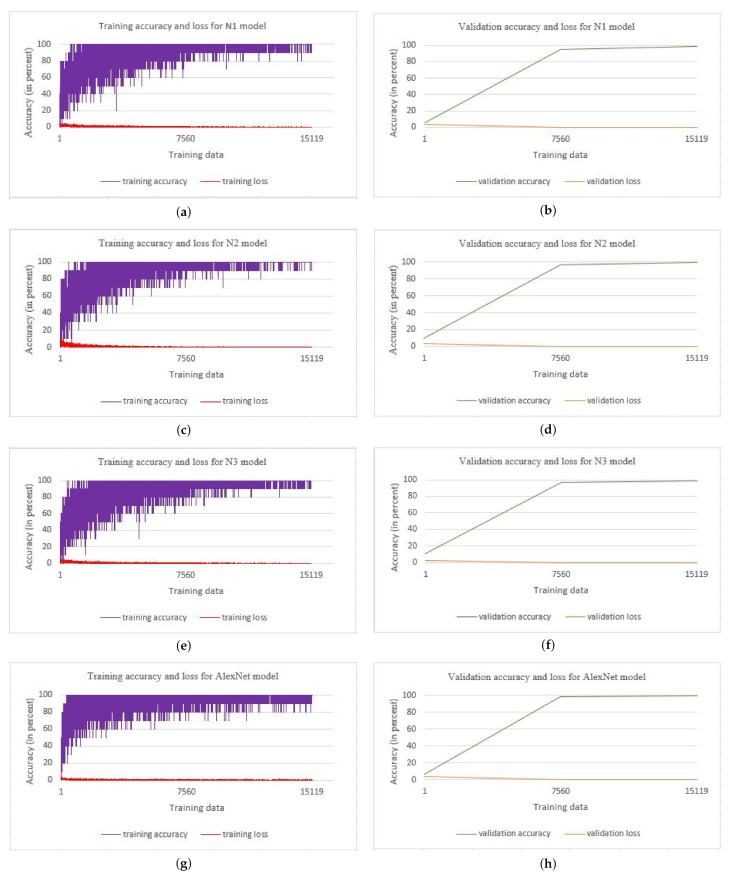
Training accuracy and training loss along with validation accuracy and validation loss for the N1 model, N2 model, N3 model, and AlexNet.

Overfitting occurs when your model fits well on the training data, but it does not generalize well on new, unseen data. Overfitting problem can be prevented by taking measures such as data augmentation, simplifying the models, using dropout, regularization, and early stopping [39,44,45]. In this work, we have used two epochs for training the model. The learning rate for the model is 0.0001. The training accuracy and training loss, along with the validation accuracy and validation loss, is as shown in Figure 8. The model with increasing training accuracy and validation accuracy and also decreasing training loss and validation loss shows that overfitting is prevented. The training accuracy and training loss are shown in Figure 8, (a) N1 model, (c) N2 model, (e) N3 model, and (g) AlexNet model. The validation accuracy and validation loss are shown in Figure 8, (b) N1 model, (d) N2 model, (f) N3 model, and (h) AlexNet model.

The comparison of models in terms of accuracy and size of the models with existing models are shown in Table 5. Jeon and Rhee [46] achieved an accuracy of 99.60% with GoogLeNet. Kaya et al. [47] in their work of plant classification, used PV and Flavia dataset with AlexNet and VGG16 models. Wang and Wang [48] classified plants with an accuracy of 84.47% with VGG16 and ResNet50 with 92.64%. For the VGG16 and VGG 19 models, the accuracy achieved by of models is 81.3% and 96.25%, respectively [49]. The combination of pruning and post-quantization was applied to VGG16, AlexNet, and LeNet model [50]. The pruning step was responsible for reducing the model size. The performance of models is 91.49%, 96.59%, and 95.2%, respectively. The ten-layer CNN model by [29] achieved an accuracy of 87.92% with the Flavia dataset and 84.02% with the PV dataset. The accuracy of the proposed N1 model is 99.45%, proposed N2 model is 99.65%, proposed N3 model is 99.55%, and AlexNet with transfer learning is 99.73% with the models trained with ad2. The size of the proposed trained models is 14.8 MB, 29.7 MB, and 14.8 MB, respectively, for the N1 model, N2 model, and N3 model as compared to AlexNet, which is 202 MB. The proposed N1 model and N3 model are 92.67% more compact than AlexNet, and the N2 model is 85.29% compact than AlexNet showing the same range accuracy results. The time for training the N1 model and N2 model is also less. The N1 model takes around 34.58% less training time than AlexNet, and the N2 model takes around 18.25% less training time than AlexNet. N3 model takes 20.23% less training time than AlexNet.

**Figure 9:**
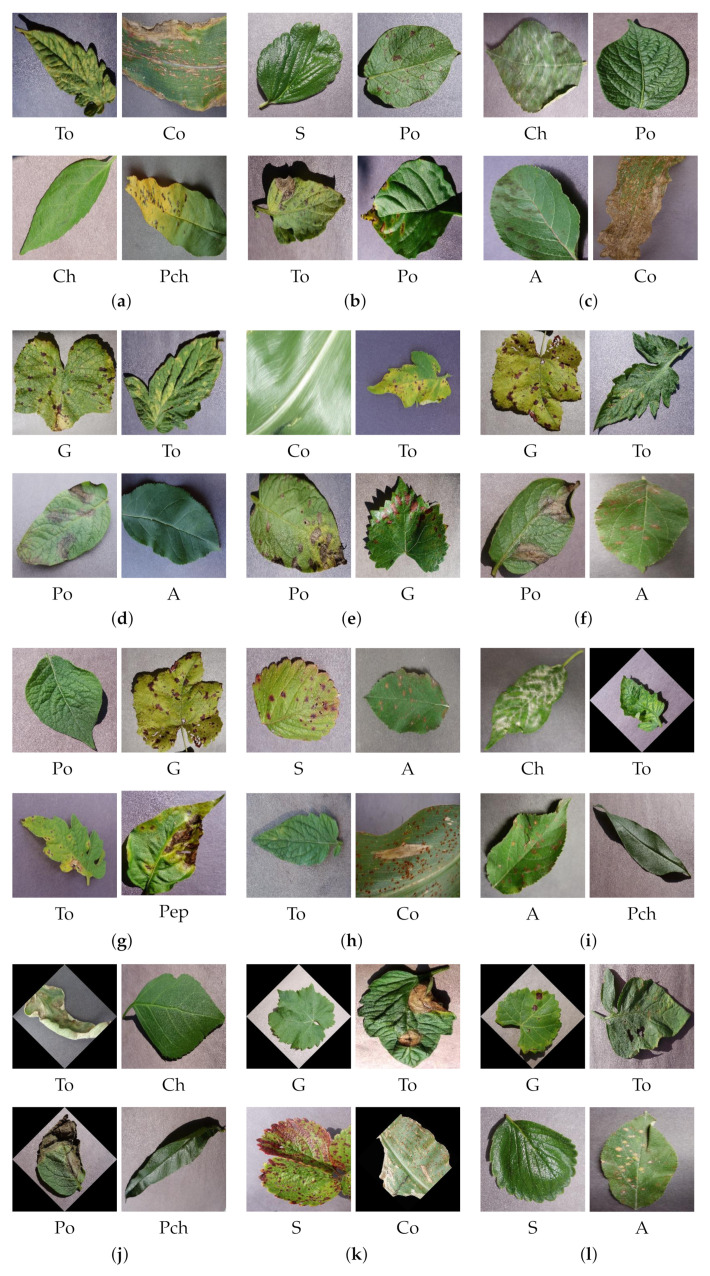
Classified output images for 80% training data with PV dataset using (**a**) N1 model with the dataset, (**b**) N2 model with the dataset, (**c**) N3 model with dataset, (**d**) AlexNet model with the dataset, (**e**) N1 model with ad1, (f) N2 model with ad1, (**g**) N3 model with ad1, (**h**) AlexNet model with ad1, (**i**) N1 model with ad2, (**j**) N2 model with ad2, (**k**) N3 model with ad2, (**l**) AlexNet with ad2.

The classified output images for the proposed N1 model, N2 model, N3 model, and AlexNet with transfer learning with 80% of training data for the PV dataset images are shown in Figure 9. The models are trained with a dataset, ad1, and ad2 and their classified output is shown here. The classified output for the proposed models and AlexNet model is shown in Figure 9, (a) N1 model with the dataset, (b) N2 model with the dataset, (c) N3 model with dataset, (d) AlexNet model with the dataset, (e) N1 model with ad1, (f) N2 model with ad1, (g) N3 model with ad1, (h) AlexNet model with ad1, (i) N1 model with ad2, (j) N2 model with ad2, (k) N3 model with ad2, (l) AlexNet with ad2. The abbreviations used for the classified output images for the PV dataset are mentioned at the start of the Results and Discussion section.

**Figure 10:**
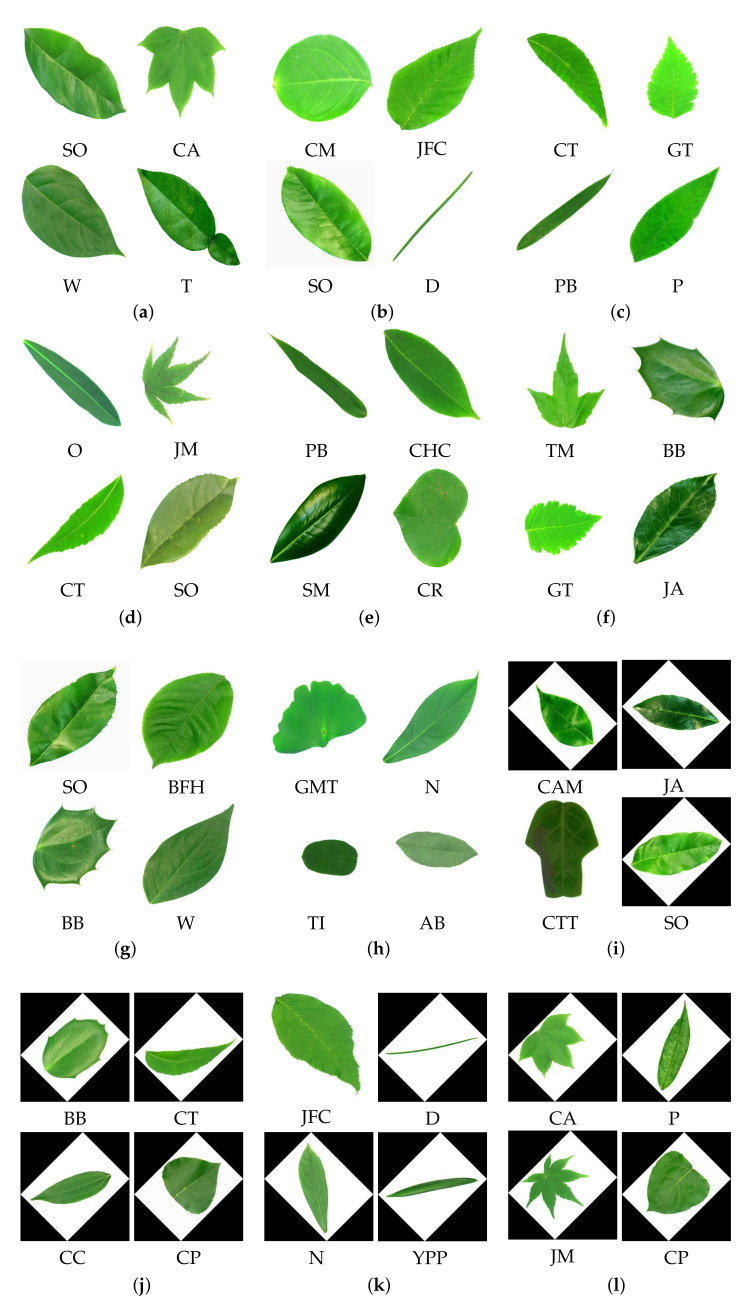
Classified output images for 80% training data with Flavia dataset using (**a**) N1 model with the dataset, (**b**) N2 model with the dataset, (**c**) N3 model with dataset, (**d**) AlexNet model with the dataset, (**e**) N1 model with ad1, (**f**) N2 model with ad1, (**g**) N3 model with ad1, (**h**) AlexNet model with ad1, (**i**) N1 model with ad2, (**j**) N2 model with ad2, (**k**) N3 model with ad2, (**l**) AlexNet with ad2.

The classified output images for the proposed N1 model, N2 model, N3 model, and AlexNet with transfer learning with 80% of training data for the Flavia dataset images are shown in Figure 10. The models are trained with a dataset, ad1, and ad2 and their classified output is shown here. The classified output for the proposed models and AlexNet model is shown in Figure 10, (a) N1 model with the dataset, (b) N2 model with the dataset, (c) N3 model with dataset, (d) AlexNet model with the dataset, (e) N1 model with ad1, (f) N2 model with ad1, (g) N3 model with ad1, (h) AlexNet model with ad1, (i) N1 model with ad2, (j) N2 model with ad2, (k) N3 model with ad2, (l) AlexNet with ad2. The abbreviations used for the classified output images for the Flavia dataset are mentioned at the start of the Results and Discussion section.

The performance of the classification by the models trained with a dataset, ad1, and ad2, is evaluated on the PV dataset by confusion matrix as shown in Table 6 for the proposed N1 model, N2 model, N3 model, and AlexNet. The confusion matrix shows the information about classification and misclassification by the model. The diagonal elements show the correct classification, and the non-diagonal elements show the misclassification information. Table 6a shows the confusion matrix for the proposed N1 model trained for 80% dataset and tested for 20% of a dataset remaining. The diagonal elements show the correct classification of each class, and cells are colored in yellow.

The effect of data augmentation on the confusion matrix of N1, N2, N3, and AlexNet models with ad1 and ad2 is shown in Table 6b. The negative number shows a decrease in classification, and a positive sign indicates an increase in classification. The cell colored in green shows the increase in classification accuracy (percentage), and the cell colored in grey shows misclassification (percentage) after data augmentation.

It is seen that the performance of the model is improved with the models trained with an augmented dataset. The accuracy of the proposed N1 model for the PV dataset is improved by 7.9% and 14.7% for the “Ch” class for the model trained with ad1 and ad2, respectively. The “Pep” class accuracy is improved by 10% and 24.3% for the model trained with ad1 and ad2, respectively. The performance of the proposed N2 model is improved by 11.3% and 21% for the “Pep” class for a model trained with ad1 and ad2, respectively. The accuracy of the N2 model is improved by 2% and 6% with a model trained with ad1 and ad2, respectively, for the “To” class. The accuracy of the proposed N3 model is improved by 8.3% and 11% for the “Po” class for the model trained with ad1 and ad2, respectively. The “Pch” class accuracy is improved by 8.9% with ad2. The performance of the AlexNet model is improved by 1.3%for the “Pch” class for a model trained with ad1. In the case of AlexNet trained with ad2, the accuracy of two classes is reduced viz. “A” and “S.” The accuracy performance for the proposed developed network N1 model, N2 model, and N3 model is seen to be improved for each of the classes with ad2.

The classification performance of the models trained with a dataset, ad1, and ad2 is evaluated on the Flavia dataset by confusion matrix as shown in Table 7 for the proposed N1 model. Table 7a shows the confusion matrix for the proposed N1 model trained for 80% dataset and tested for 20% of a dataset remaining. The diagonal elements show the correct classification of each class, and cells are colored in yellow. The effect of data augmentation on the confusion matrix of N1, N2, N3, and AlexNet models with ad1 and ad2 is shown in Table 7b. The negative number shows a decrease in classification, and a positive sign indicates an increase in classification. The cell colored in green shows the increase in classification accuracy (percentage), and the cell colored in grey shows misclassification (percentage) after data augmentation.

The accuracy of the proposed N1 model for the Flavia dataset is improved by 5% and 12.4% for the “CR” class for the model trained with ad1 and ad2, respectively, and 10.8% and 16% for the proposed N2 model with ad1 and ad2. The “CT” class accuracy is improved by 7.5% with ad1 and 20.4% with ad2 for the proposed N1 model. The performance of the N2 model is improved by 7% and 13% for the “GMT” class for a model trained with ad1 and ad2, respectively. The accuracy of the proposed N3 model is improved by 19.3% for the “CC” class for the model trained with ad2. The performance of the AlexNet model is improved by 5.8% and 5.7% for the “YPP” class for a model trained with ad1 and ad2, respectively.

Data augmentation influences the average precision of the class [53,54]. Based on the confusion matrix, the performance parameters of macro recall, macro precision, macro F1 score, and mean accuracy are evaluated for the PV and Flavia datasets. The performance parameters of the proposed N1 model, N2 model, N3 model, and AlexNet is shown in Table 8. The performance parameters of macro recall, macro precision, macro F1 score, and mean accuracy for the PV and Flavia dataset are compared here for data, ad1 and ad2. It is seen that the performance parameters are improved with the ad2. The proposed developed N1, N2, and N3 models have the same range performance as AlexNet. The size of these models is much more compact to AlexNet and gives great results.

For analyzing the results of the experimental designs by statistical tests, an analysis of variance (ANOVA) is developed by [55]. The ANOVA is performed on the performance parameters for the proposed models, and AlexNet trained with the dataset, ad1, and ad2 of both the datasets is shown in Table 9, Table 10 and Table 11. The parameters evaluated are Sum of Squares (SS), degree of freedom (df), mean squares (MS), *p*-value, F value, and F critical value. The condition for statistical significance is evaluated based on the *p*-value and if the F value is less than the F critical value. If the *p*-value is between 0.0001 to 0.001, then it is extremely statistically significant when the *p*-value is between 0.001 to 0.01, then it is very statistically significant when the *p*-value is between 0.01 to 0.05, then it is statistically significant, and when the *p*-value is greater than 0.05, then there is no statistical significance. In all three tables for ANOVA, we can see the statistical significance for the models evaluated on both datasets.

The ability of the trained model to classify new data is an important factor in decision making. The PV dataset has nine classes belonging to nine plant species. The classification of plant species for PV dataset images that were not part of the training and testing dataset is completed. The validation accuracy of the N1 model, N2 model, N3 model, and AlexNet models trained with PV dataset is shown in Table 12. The images that were not part of the training and testing dataset are used for validation of the model into respective species. The validation performance of pepper is lower compared to other species. The proposed N2 model classifies the apple species with 92.5% accuracy, N2 model and AlexNet model classify cherry with 95% accuracy. The validation accuracy of the N2 model and AlexNet is 97.5% for the corn. The validation of grape, peach, and strawberry is good for all the models. The validation accuracy of the N3 model for tomato is 91.11%. Overall, the performance of the N2 model is more as compared to N1 and N3 models.

The classification of plant species for Flavia dataset images that were not part of the training and testing dataset is completed. In the case of the Flavia dataset, each of the 32 classes belong to different plant species. Validation accuracy of proposed N1 model, N2 model, N3 model, and AlexNet models trained with Flavia dataset is shown in Table 13. Almost all the species are showing good classification except for the Cam class. Overall, the N2 model is performing equally well as AlexNet. N2 model achieves better performance in classification, as well as validation for PV and Flavia dataset with compact model size.

## 4. Performance of Proposed Models for the Classification of Tomato Plant Disease

**Figure 11:**
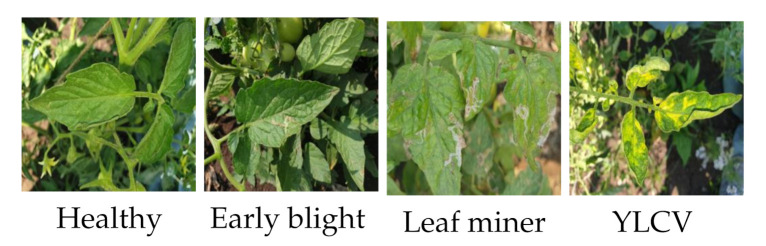
Plant leaf images of Lavale data.

The proposed model performed well in the classification of plant leaves. The proposed models are further used for classifying the disease in tomato plant leaf. The image data were collected from a tomato farm from Lavale, Pune, India. These data were captured with a mobile phone camera with the specification Super Speed Dual Pixel 12MP AF sensor. The images were captured with background around the tomato plant leaves. In real-life applications, input images cannot be expected to be of high quality. The images captured for each of the four classes were 300, which were augmented to 126,000 images. The augmented images are used to train the proposed models and AlexNet. The data of tomato plants are tomato early blight, leaf miner, and yellow leaf curl virus (YLCV) disease classes, along with healthy leaves. Figure 11 shows the tomato plant disease and a healthy class of Lavale farm dataset.

**Figure 12:**
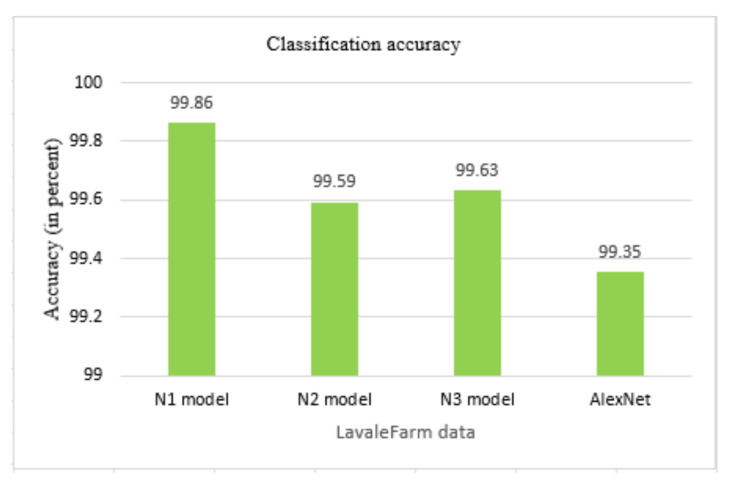
Classification accuracy of models for Lavale farm dataset.

The classification accuracy of the proposed models and AlexNet is shown in Figure 12. The proposed N1 model achieves an accuracy of 99.864%, N2 achieves 99.59%, N3 achieves 99.63%, and AlexNet achieves an accuracy of 99.35%. The proposed N1 model takes 22.82%, N2 model takes 56.18%, and N3 model takes 25.73% less training time than AlexNet model. The N1 and N3 model are 99.06% and N2 model is 98.11% compact than AlexNet model. The proposed model’s performance shows that they are capable of classifying the tomato plant disease images with complex backgrounds with good accuracy, less training time and compact model size. The proposed model’s size is compact and takes less training time than the state of art models. The proposed models can be deployed as a stand-alone mobile app that will benefit the farmer with its results.

## 5. Conclusions

The classification of Plant leaf images of nine classes of the PV database using proposed CNN models viz N1 model, N2 model, N3 model, and AlexNet with transfer learning is performed in this work. The developed model shows better performance after data augmentation is applied to them. The accuracy achieved by the proposed developed models is 99.45% with N1 model, 99.65% with N2 model, 99.55% with N3 model, and 99.73% with the AlexNet model for the PV dataset. The accuracy achieved by the proposed developed models is 99.17% with N1 model, 99.59% with N2 model, 99.36% with N3 model, and 99.87% with the AlexNet model for the Flavia dataset with 32 classes. The accuracy of the developed models is equally good as AlexNet. The proposed N1 model and N3 model are 92.67% compacts than AlexNet, and N2 model is 85.3% compact than AlexNet. The training time of the developed model is reduced by 34.58% for the N1 model and 18.25% for the N2 model, and 20.23% for N3 as compared to the AlexNet. The N2 model has a compact size as compared to AlexNet and shows the same range accuracy. The classification of species is done by these trained models on the different images from PV and Flavia datasets that are not included in training and testing the models. The overall performance of the N2 model is more than N1 and N3 models. The experiments on two challenging datasets of PV and Flavia confirm the effectiveness of our method. The distinctiveness of proposed model classifies diseased plant leaves in the images captured with a mobile phone. The proposed models can be deployed as a stand-alone mobile app that will benefit the farmers as the proposed models are compact and give good classification results. The automatic plant classification will help in plant management thereby benefiting the society.

## Figures and Tables

**Table 1:** A comparative analysis of the work related to the classification of plants.

Ref. No.	Objective	Dataset	Number of Classes	Model	Accuracy
Dyrmann et al. [24]	Plant leaf classification	Six different datasets	22	CNN	86.20%
Mohanty et al. [23]	Identify 14 crop species	PlantVillage	38	AlexNet	99.27%
			38	GoogLeNet	99.34%
Barré et al. [25]	plant identification system	LeafSnap	184	LeafNet	86.30%
			60	LeafNet	95.80%
			32	LeafNet	97.90%
Lee et al. [10]	Plant leaf classification	Malayakew	44	Deep CNN (D1) MLP	97.70%
			44	Deep CNN (D1) SVM (linear)	98.10%
Gao et al. [1]	Leaf Identification	LifeCLEF 2015	30	3SN	84.20%
Dileep and Pournami [28]	Medicinal plant classification	AyurLeaf	40	AlexNet	94.87%
			40	Ayurleaf CNN	95.06%
Duong-Trung et al. [12]	Medicinal plant classification	Own data	20	MobileNet	98.50%
Liu et al. [29]	Classification of 32 different plant leaves	Flavia	32	Ten-layer CNN model	87.92%
Bodhwani et al. [9]	Plant Identification	LeafSnap	180	ResNet	93.09%
Tiwari [5]	Plant leaf classification	Dataset collected by [30]	30	DNN	91.17%
			30	CNN	95.58%
Yang et al. [6]	Classification of plant leaf	Own data	15	VGG16	91.50%
			15	VGG19	92.40%
			15	Inception- ResNetV2	89.60%
Villaruz [14]	Identification of berry plants	Own data	3	AlexNet	97.80%

**Table 2:** A comparative analysis of the work related to the classification of plants disease.

Ref. No.	Model	Dataset	Objective	Future Scope
Wang et al. [16]	VGG16	PV	Apple black rot disease severity	More data at various stages of disease can be used to improve accuracy.
Mohanty et al. [23]	AlexNet, GoogLeNet	PV	Identify 14 crop species and 26 diseases	Image data from a smartphone can be supplemented with location and time information to improve accuracy even further.
Brahimi et al. [31]	AlexNet, GoogLeNet	PV	Tomato plant disease classification	Reduce the computation and the size of deep models
Bharali et al. [32]	CNN model	Google images	Classifying into healthy and disease class for different plant	Larger datasets and more complex networks can be created to assess performance and improve accuracy.
Ahmad et al. [33]	VGG16, VGG19, ResNet Inception-V3	Own data	Classification of Tomato plant disease	Optimize these models for better performance on real-world field-based data.
Anadhakrishnan et al. [34]	AlexNet, VGG16, LeNet, ResNet, CNN model	PV	Classification of Tomato plant disease	Improve computational time
Oyewola et al. [36]	deep residual neural network	Cassava Disease Dataset from Kaggle	cassava mosaic disease classification	Novel image augmentation methods combined with other deep neural networks to improve accuracy
Alli et al. [37]	MobileNetV2	own data	Cassava disease recognition	multi-class detection for identifying a variety of plant diseases
Almadhor et al. [35]	Bagged Tree classifier	own data	Guava fruit disease detection	employing deep learning methods to extract features automatically
Kundu et al. [38]	Custom-Net	own data	pearl millet disease classification	scope of making the predictions based on the parametric dataset collected by the data collector part

**Table 3:** The amalgamation used to augment the dataset.

**Augmentation 1 (ad1)**				
**Noise**	Salt and pepper noise			
**Blur**	Gaussian blur			
**Position augmentation**	Random scaling	random rotation		
**Augmentation 2 (ad2)**				
**Position augmentation**	45° Rotation	135° Rotation	225° Rotation	315° Rotation
	Horizontal flip	Vertical flip		
**Color augmentation**	Hue	Saturation	Contrast	

**Table 4:** Convolution layers for N1 model, N2 model, and N3 model.

CNN Layer	N1 Model	N2 Model	N3 Model
1st Conv2D	3 × 3, 8	3 × 3, 16	7 × 7, 8
Maxpooling stride	2	2	2
2nd Conv2D	3 × 3, 16	3 × 3, 32	5 × 5, 16
Maxpooling stride	2	2	2
3rd Conv2D	3 × 3, 32	3 × 3, 64	3 × 3, 32

**Table 5:** Performance comparison of proposed work with other existing work in plant classification.

Source	Dataset	Method	Accuracy	Size
Mohanty et al. [23]	PV	AlexNet	99%	227 MB [51]
		GoogLeNet	99%	27 MB [51]
Lee et al. [10]	Flavia	AlexNet	99.40%	202 MB [51]
Jeon and Rhee [46]	PV	GoogLeNet	99.60%	27 MB [51]
Kaya et al. [47]	Flavia	Alexnet	97.89%	202 MB [51]
		VGG16	98.16%	515 MB [51]
	PV	Alexnet	98.6%	202 MB [51]
		VGG16	99.8%	515 MB [51]
Wang [48]	Flavia	VGG16	84.47%	515 MB [51]
		ResNet50	92.24%	96 MB [51]
Anubha Pearline et al. [49]	Flavia	VGG16	95%	515 MB [51]
		VGG19	96.25%	535 MB [51]
Venkatesh et al. [52]	PV	VGG16	81.3%	515 MB [51]
		VGG16 +Inception	92.2%	-
Fountsop et al. [50]	Flavia	VGG16 Pruning +	91.49%	36.76 MB
		post-quantization		
		AlexNet Pruning +	96.59%	32.37 MB
		post-quantization		
		LeNet Pruning +	95.02%	9.91 MB
		post-quantization		
Liu et al. [29]	Flavia	ten-layer CNN	87.92%	7 MB
**Proposed work**	PV	**N1 model**	99.45%	14.8 MB
		**N2 model**	99.65%	29.7 MB
		**N3 model**	99.55%	14.8 MB
		**AlexNet**	99.73%	202 MB
	Flavia	**N1 model**	99.17%	14.8 MB
		**N1 model**	99.59%	29.7 MB
		**N1 model**	99.36%	14.8 MB
		**AlexNet**	99.87%	202 MB

**Table 6:** Confusion matrix for proposed model for PV dataset.

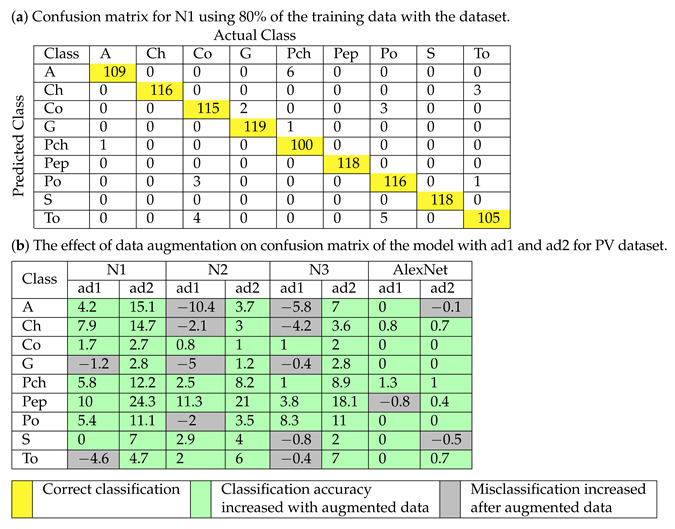

**Table 7:** Confusion matrix for proposed model for Flavia dataset.

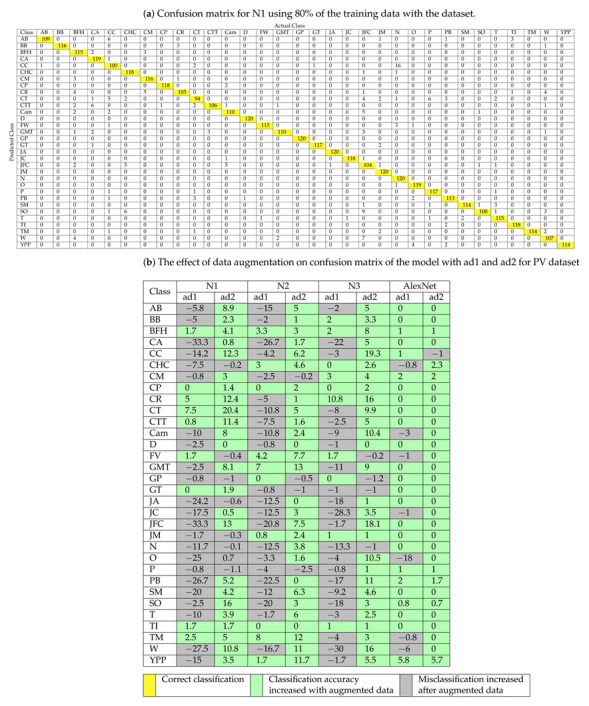

**Table 8:** Performance parameters of N1 model, N2 model, N3 model, and AlexNet trained with data, ad1, and ad2.

Dataset	Model	Data	Macro	Macro	Macro	Mean
			Recall	Precision	F1_score	Accuracy
PV	N1	data	87.76%	86.58%	86.57%	99.16%
		ad1	89.50%	89.31%	89.26%	99.33%
		ad2	99.45%	99.45%	99.45%	99.97%
	N2	data	92.35%	92.03%	91.95%	99.50%
		ad1	91.54%	90.83%	90.83%	99.43%
		ad2	99.65%	99.65%	99.65%	99.98%
	N3	data	90.20%	89.61%	89.57%	99.35%
		ad1	90.32%	89.80%	89.71%	99.36%
		ad2	99.55%	99.55%	99.55%	99.97%
	AlexNet	data	98.60%	98.53%	98.52%	99.91%
		ad1	98.99%	98.98%	98.98%	99.94%
		ad2	99.74%	99.73%	99.73%	99.98%
Flavia	N1	data	94.54%	94.30%	94.30%	99.64%
		ad1	86.43%	85.63%	85.45%	99.10%
		ad2	99.18%	99.17%	99.17%	99.95%
	N2	data	96.05%	95.91%	95.91%	99.74%
		ad1	90.49%	89.77%	89.76%	99.36%
		ad2	99.59%	99.59%	99.59%	99.97%
	N3	data	93.99%	93.83%	93.80%	99.61%
		ad1	88.49%	87.99%	87.76%	99.25%
		ad2	99.37%	99.36%	99.36%	99.96%
	AlexNet	data	99.50%	99.48%	99.48%	99.97%
		ad1	99.02%	98.93%	98.93%	99.93%
		ad2	99.87%	99.87%	99.87%	99.99%

**Table 9:** ANOVA analysis of performance parameters evaluated for dataset.

Source of Variation	SS	df	MS	F	*p*-Value	F Critical	Significance
Dataset	77.688	1	77.688	6.659	0.0164	4.259	**
Models	191.983	3	63.994	5.485	0.0051	3.009	***
Dataset × Models	24.687	3	8.229	0.705	0.5582	3.009	NS
Within	279.985	24	11.666				
Total	574.343	31					

*** *p* < 0.001, ** *p* < 0.01, * *p* < 0.05; NS, *p* ≥ 0.05.

**Table 10:** ANOVA analysis of performance parameters evaluated for ad1.

Source of Variation	SS	df	MS	F	*p*-Value	F Critical	Significance
Dataset	12.276	1	12.276	0.604	0.4446	4.259	NS
Models	366.819	3	122.273	6.015	0.0033	3.009	***
Dataset × Models	7.659	3	2.553	0.125	0.944	3.009	NS
Within	487.848	24	20.327				
Total	874.602	31					

*** *p* < 0.001, ** *p* < 0.01, * *p* < 0.05; NS, *p* ≥ 0.05.

**Table 11:** ANOVA analysis of performance parameters evaluated for ad2.

Source of Variation	SS	df	MS	F	*p*-Value	F Critical	Significance
Dataset	4.43 × $10^{-6}$	1	4.43 × $10^{-6}$	0.816	0.3752	4.259	NS
Models	6.23 × $10^{-5}$	3	2.08 × $10^{-5}$	3.829	0.0225	3.009	*
Dataset × Models	1.13 × $10^{-5}$	3	3.77 × $10^{-6}$	0.696	0.5634	3.009	NS
Within	0.0001	24	5.42 × $10^{-6}$				
Total	0.0002	31					

*** *p* < 0.001, ** *p* < 0.01, * *p* < 0.05; NS, *p* ≥ 0.05.

**Table 12:** Validation accuracy of proposed N1 model, N2 model, N3 model, and AlexNet models trained with PV dataset.

Species	N1	N2	N3	AlexNet
“Apple”	82.5%	92.5%	90%	100%
“Cherry”	75%	95%	85%	95%
“Corn”	95%	97.5%	90%	97.5%
“Grape”	90%	97.5%	95%	100%
“Peach”	80%	85%	90%	100%
“Pepper”	40%	60%	70%	100%
“Potato”	73.33%	90%	86.67%	100%
“Strawberry”	100%	90%	100%	100%
“Tomato”	88.89%	84.44%	91.11%	95.56%

**Table 13:** Validation accuracy of N1 model, N2 model, N3 model, and AlexNet models trained with Flavia dataset.

Species	N1	N2	N3	AlexNet
AB	100%	60%	80%	100%
BB	100%	100%	100%	100%
BFH	100%	100%	100%	100%
CA	100%	100%	100%	100%
Cam	10%	10%	10%	50%
CC	60%	50%	50%	100%
CHC	80%	90%	90%	100%
CM	100%	100%	100%	100%
CP	100%	100%	100%	100%
CR	90%	80%	90%	100%
CT	30%	30%	20%	80%
CTT	100%	100%	100%	100%
D	90%	100%	90%	100%
FW	90%	90%	90%	100%
GMT	100%	100%	100%	100%
GP	100%	100%	100%	100%
GT	80%	50%	70%	100%
JA	100%	100%	100%	100%
JC	50%	50%	60%	80%
JFC	50%	60%	60%	100%
JM	100%	100%	100%	100%
N	90%	90%	90%	100%
O	100%	100%	100%	100%
P	100%	100%	100%	100%
PB	50%	70%	60%	90%
SM	40%	70%	40%	100%
SO	70%	60%	70%	90%
T	70%	90%	90%	100%
TI	90%	100%	90%	100%
TM	90%	80%	90%	100%
W	90%	90%	90%	100%
YPP	30%	90%	20%	70%

## Data Availability

Not applicable.
